# Cultural adaptation and psychometric validation of the Gastrointestinal Unhelpful Thinking Scale

**DOI:** 10.3389/fmed.2026.1807329

**Published:** 2026-03-23

**Authors:** Changqin Xu, Yaocheng Shi, Weiqin Huang, Huiren Zhuang, Lili Ma

**Affiliations:** 1 Department of Nursing, School of Medicine, Shanghai East Hospital, Tongji University, Shanghai, China; 2 Department of Dental VIP Clinic, School of Medicine, Shanghai East Hospital, Tongji University, Shanghai, China

**Keywords:** cross-cultural adaptation, Gastrointestinal Unhelpful Thinking Scale, C-GUTs, irritable bowel syndrome, psychometric validation

## Abstract

**Background:**

Gastrointestinal symptoms are often influenced by cognitive and emotional factors. The Gastrointestinal Unhelpful Thinking Scale (GUTs) was developed to assess such cognition in patients with functional gastrointestinal disorders. However, no validated Chinese version exists by now.

**Aim:**

To conduct translation, cultural adaption and psychometric evaluation of the Chinese version of the GUTs amongst patients with irritable bowel syndrome (IBS).

**Methods:**

Following the Brislin translation model for cross-cultural adaptation, we recruited 212 IBS patients from a tertiary hospital in Shanghai using convenience sampling. The validation process included assessment of content validity, construct validity and reliability.

**Results:**

The Chinese GUTs demonstrated excellent content validity, with the content validity level and the average level of scale being 0.80 and 0.987, respectively. Exploratory factor analysis verified the original two-factor structure (pain catastrophizing and visceral sensitivity), accounting for 66.240% of the total variance. Confirmatory factor analysis indicated the model fit was acceptable (χ^2^/df = 2.991, CFI = 0.853, RMSEA = 0.038). The scale demonstrated good internal consistency (Cronbach’s α = 0.947) and test-retest reliability (ICC = 0.844). Spearman-Brown coefficient was 0.912, and considered as strong split-half reliability.

**Conclusion:**

The translated GUTs shows robust psychometric properties and is a valid, reliable scale for assessing gastrointestinal-specific unhelpful thinking patterns in Chinese IBS patients, suitable for clinical applications.

## 1 Introduction

Irritable bowel syndrome (IBS) is one of the most prevalent functional gastrointestinal disorders (FGID) and a classic disorder of gut-brain interaction (DGBI). It is characterized by recurrent abdominal pain associated with defecation or changes in bowel habits, without detectable structural abnormalities (1). Its global prevalence is estimated to be approximately 5%, with varying rates across different countries and regions (2). In China, the reported prevalence of IBS is around 4.4%, and it shows an upward trend, posing significant threats to the quality of life of patients and imposing a heavy burden on the healthcare system (3). The chronic and recurrent nature of IBS, along with symptoms such as abdominal pain, bloating, and altered bowel habits, significantly affects patients’ daily activities, work efficiency, and psychological health (4).

The pathophysiology of IBS is multifactorial and not fully elucidated, but it is widely acknowledged as a biopsychosocial condition. Studies have highlighted the crucial role of the “microbiota - gut - brain axis,” a complex, bidirectional communication network linking the central nervous system, the enteric nervous system, and the gut microbiota (5, 6). Within this context, psychological factors are not merely outcomes of chronic symptoms but are essential to the onset, exacerbation, and perpetuation of IBS. A significant amount of evidence confirms a high comorbidity between IBS and psychological distress, especially anxiety and depression (7–9). It is reported that 40%–50% of irritable bowel syndrome patients suffer from psychological disorders such as anxiety, depression, and somatization of mental symptoms, while 80% of irritable bowel syndrome patients experience exacerbations and episodes closely associated with psychological factors (10). This intricate relationship emphasizes the necessity of incorporating psychological assessment into the comprehensive management of IBS (11). Among the various psychological mechanisms, two processes have emerged as particularly prominent in IBS: visceral sensitivity and pain catastrophizing. Visceral sensitivity (VS), also known as gastrointestinal - specific anxiety (GSA), refers to thoughts, feelings, and behaviors from the fear of GI (gastrointestinal) symptoms/sensations and related contexts. Patients with high VS may experience hypervigilance, fear, anxiety, or avoidance in relation to GI symptoms/sensations and associated contexts (e.g., restaurants, places with restricted toilet access). They may also have an enhanced perception of GI-related somatic sensations (12). Pain catastrophizing (PC), a concept from the broader pain literature, describes an individual’s tendency to overestimate the threat of pain, ruminate on its consequences, and feel helpless when in pain (13). In the context of IBS, PC intensifies the perceived severity and distress related to abdominal pain, contributing to greater functional impairment and increased healthcare - seeking behavior (14). These cognitive processes represent specific, maladaptive thinking patterns that exacerbate the brain - gut axis dysfunction in IBS.

The latest Chinese expert consensus on IBS underscores the significance of an integrated, patient - centered treatment approach that tackles both gastrointestinal symptoms and associated psychosocial factors (4). To effectively adopt such an approach, healthcare providers need valid and reliable tools to identify and quantify these crucial psychological drivers. Currently, the assessment of psychological factors in Chinese IBS patients frequently depends on generic tools, such as SCL-90 (the Symptom Checklist - 90) or the Hamilton Anxiety and Depression Scales (15, 16). Although these instruments are useful for screening general distress, they are not designed to capture the gastrointestinal - specific cognitive patterns that are central to the IBS experience. The Visceral Sensitivity Index (VSI) and the Pain Catastrophizing Scale (PCS) are two well-established measures for these constructs (12, 13), yet their combined use in clinical practice can be time - consuming, potentially diminishing feasibility in busy healthcare settings. To address this gap, Knowles et al. developed and validated the GUTs (17). The GUTs is a 15 - item instrument specifically designed to concurrently assess the two core cognitive processes of VS (6 items) and PC (9 items) among patients. Its development went beyond the simple combined use of the VSI and PCS. The original validation study demonstrated a two-factor structure in both gastrointestinal and non-gastrointestinal populations. The test–retest reliability coefficient of the GUTs was 0.93, and those for the two subscales were 0.86 and 0.94, respectively (17). This makes the GUTs an efficient and targeted tool for both clinical and research settings to evaluate and monitor the cognitive-affective factors that maintain and exacerbate gastrointestinal symptoms.

However, the application of the GUTs in the Chinese population is currently impeded by the absence of a culturally and linguistically adapted version. Direct translation without a rigorous cross-cultural validation process may compromise the conceptual equivalence, content validity, and reliability of the instrument (18). Differences in illness beliefs, symptom expression, and the conceptualization of psychological distress between Western and Chinese cultures call for a systematic adaptation process to ensure that the tool is appropriate, comprehensible, and relevant for Chinese patients (19). Moreover, the lack of a validated version of the GUTs restricts its utility in clinical practice among Chinese-speaking populations, potentially leading to misdiagnosis or inadequate treatment. Therefore, developing a culturally and linguistically appropriate version of the GUTs is essential for enhancing the quality of care and improving patient outcomes in Chinese healthcare settings.

Therefore, the objectives of this study were: (1) to translate and culturally adapt the original GUTs into Chinese following established guidelines, and (2) to comprehensively evaluate the psychometric properties, including validity and reliability, of the Chinese version of GUTs in Chinese patients diagnosed with IBS. The translation, adaptation, and validation of the Chinese GUTs will offer health practitioners in China a valuable, standardized tool, thus facilitating targeted psychological interventions and ultimately enhancing patient outcomes.

## 2 Materials and methods

### 2.1 Study design

The study obtained authorization from the developers of the GUTS scale and translated the original version of GUTs into Chinese according to Brislin translation model (20). To conduct cultural adaptation and validation, we employed a cross-sectional study design, focusing on two key tasks: translation and cross-cultural adaptation, as well as psychometric validation of the scale. This study was followed the STROBE checklist.

#### 2.1.1 Phase I translation and cultural adaptation

The original GUTs, developed by Knowles et al. (17), is a 15-item scale designed to evaluate two key cognitive processes in gastrointestinal disorders. It comprises two subscales: PC and VS. PC is composed of nine items and VS is composed of six items. All items are rated on a five-point Likert scale, while 0 represents “Strongly disagree” and 5 represents “Strongly agree.” Subscale scores were obtained by calculating the average score of each item. The total GUTs score is derived from the average of the two subscale. Higher scores indicate more severe levels of gastrointestinal-focused unhelpful thinking. The exploratory factor analysis revealed that χ^2^ was 2.08, the Tucker–Lewis Index was 0.94, the comparative fit index was 0.96, the standardized root mean square residual was 0.05, and the root mean square error of approximation was 0.07 (17). The test-retest reliability of the GUTs (the total scale and two subscales) was 0.93, 0.86, and 0.94, respectively (17).

**FIGURE 1:**
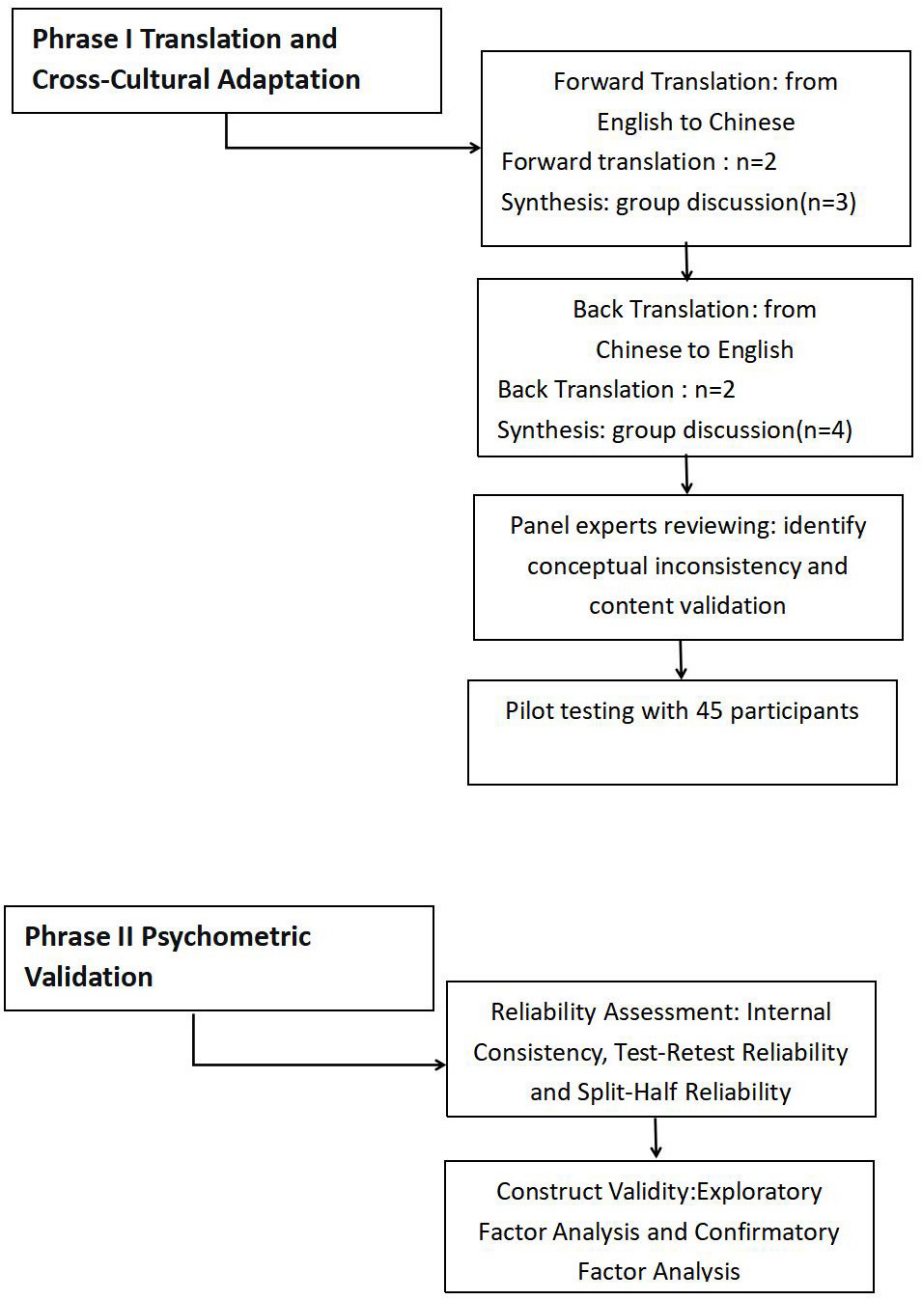
Flowchart of the translation and measurement property assessment of the Gastrointestinal Unhelpful Thinking Scale (GUTs).

The translation and cultural adaptation of GUTs follows the well-established Brislin translation model (20) and align with international guidelines for cultural adaptation of self-report scales (18). This process encompasses four key stages, as illustrated in Figure 1.

#### 2.1.2 Step 1: forward translation

The original English GUTs was translated the into Chinese (Mandarin) by two bilingual translators independently whose native language was Chinese. Translator 1 (T1) was a healthcare professional with over 10 years of experience in gastroenterology, aware of the concepts being examined. Translator 2 (T2) was a professional translator unfamiliar with the instrument’s specific constructs, aiming to capture natural and colloquial language. This resulted in two initial Chinese versions (T1 and T2). The two forward translations (T1 and T2) and the original instrument were reviewed by a third bilingual researcher. By group discussion, discrepancies in wording, conceptual meaning, and cultural relevance were discussed until a consensus was reached, resulting in a synthesized Chinese version (T3).

#### 2.1.3 Step 2: back translation

The T3 version was translated from Chinese into English by two other bilingual translators independently, both native English speakers were blinded to the original GUTs and unfamiliar with the study’s objectives. This step aimed to identify any conceptual errors or deviations from the original meaning. Subsequently, the research team compared the two back-translated versions (BT1 and BT2) with the original GUTs. Any discrepancies were reviewed and resolved, leading to a revised Chinese version (BT3).

#### 2.1.4 Step 3: panel experts reviewing and content validation

A panel of 15 experts was convened to assess the pre-final version (BT3) for content validity and cultural equivalence. The panel included gastroenterologists (*n* = 5), clinical psychologists (*n* = 3), senior digestive specialty nurses (*n* = 5), and methodology experts in scale development (*n* = 2). Using a self-designed expert consultation form, they scored each item with a four-point scale for its clarity and relevance as well as cultural appropriateness. The item-level Content Validity Index (I-CVI) and the scale -level Content Validity Index (S-CVI) were used to calculate the Content Validity Index (21). The I-CVI with values below 0.78 was deemed for revision or elimination. The experts also provided qualitative feedback on semantic, idiomatic, experiential, and conceptual equivalence. The committee held discussions to reach a consensus on all items, producing the pre-final version for field testing.

#### 2.1.5 Step 4: pilot testing

In January 2025, purposive sampling was employed to recruit 45 IBS patients from a tertiary hospital in XX for a preliminary survey. Feedback and suggestions were recorded to revise items with unclear, difficult-to-understand, or ambiguous wording. Inclusion criteria for participants: Diagnosis confirmed with reference to the Rome IV Diagnostic Criteria for FGIDs (22): age 18 years or older; possess basic communication and comprehension abilities; written consents were acquired from patients and their guardians. Exclusion criteria: inflammatory irritable bowel syndrome; combined with other disorders such as cardiac or renal failure, tumors, progressive liver or pancreatic diseases; history of mental illness and receiving medication treatment. Sample size calculation for pilot study: according to Pilot and Beck, the number of pilot tests should be 3–4 times of the number of total items (23). The compiled GUTs version contains 15 items, then the sample size should be 45 cases.

#### 2.1.6 Phase II psychometric validation

A cross-sectional study was conducted to validate the psychometric evaluation of the GUTs, which was completed in two steps. Internal Consistency, Test-Retest Reliability and Split-Half Reliability were tested for reliability. And Exploratory Factor Analysis (EFA) and Confirmatory Factor Analysis (CFA) were used for construct validity. The samples for EFA and CFA required at least 100 and 200 cases (24). The inclusion criteria and exclusion criteria were equal to those in the pilot study.

#### 2.1.7 Step 1: construct validity

Construct validity was evaluated through EFA and CFA. EFA was conducted on the initial sample to explore the underlying factor structure of the scale. The suitability of the data for factor analysis was first assessed using the Kaiser-Meyer-Olkin (KMO) measure of sampling adequacy and Bartlett’s test of sphericity (25). A KMO value greater than 0.6 and a significant Bartlett’s test (*p* < 0.05) were considered indicative of appropriate data for EFA (25). CFA was used to validate the factor structure obtained from EFA. The goodness-of-fit indices, comparative fit index (CFI), Tucker-Lewis index (TLI) and RMSEA were used to assess the model fit (25).

#### 2.1.8 Step 2: reliability assessment

Cronbach’s α was assessed for Internal consistency (≥0.70 acceptable). Intraclass correlation coefficient was conducted for Test-retest reliability (ICC > 0.75 indicating good reliability). Time interval between test-retest was set at 2 weeks to minimize the potential influence of external factors on participants’ responses. Spearman coefficient and Guttman coefficient were conducted for Split-half reliability (26, 27).

### 2.2 Data collection and procedures

This study was conducted at the gastroenterology outpatient department of a comprehensive tertiary hospital in XX, China, between February 2025 and June 2025. The research team distributed the paper scale or the electronic scale through cell phone scanning. Prior to data collection, the research team provided detailed instructions on how to complete the scale. Written consents were acquired before the participant filled out the investigation forms. Each Participant was provided sufficient time to fill out the scale independently, and the research team was available to answer any questions or concerns that arose during the process. After completion, the scales were collected and checked for completeness and accuracy.

### 2.3 Statistical analysis

IBM SPSS Statistics 25.0 and AMOS 24.0 were used for data analysis. Descriptive statistics, including means, standard deviations, frequencies, and percentages, were computed to summarize the demographic characteristics of the participants and the item-level scores of the scale. The content validity was determined by assessing the relevance and comprehensiveness of the scale, which was calculated with I-CVI and S-CVI (with I-CVI validity being no less than 0.78 and S-CVI validity no less than 0.5) (28). EFA and CFA were used to examine the scale structure. *P* < 0.05 was considered as statistical significance.

### 2.4 Ethical consideration

Ethical approval was obtained from Hospital’s Ethics Committee [(2025) Research Approval No. 151]. Potential participants were identified by their visiting gastroenterologists during routine consultations. Written consents were acquired from all the participant before they filled out the investigation forms in the study. The study strictly adhered to the principles of confidentiality, ensuring that all personal and medical information of the participants was protected and used solely for research purposes.

## 3 Results

### 3.1 Demographic characteristics of patients

A total of 212 patients with IBS was investigated, and all investigating forms were collected (collecting rate was 100%). And 47.2% participants were male. The age of the patients was mainly 31–40 years old and over 60 years old. Among them, 158 patients were single (74.5%), 42.5% of the patients had an education level of bachelor’s degree or above (90/212), and 67.9% of the patients were employed (144/212) in Table 1.

**TABLE 1:** Participant characteristics (*N* = 212).

Variables		Number	Percentage
Gender			
	Male	100	47.2
	Female	112	52.8
Age			
	18∼20 years old	4	1.9
	21∼25 years old	12	5.7
	26∼30 years old	42	19.8
	31∼40 years old	49	23.1
	41∼50 years old	31	14.6
	51∼60 years old	22	10.4
	>60 years old	52	24.5
Martial status			
	Married	54	25.5
	Single	158	74.5
Educational level			
	Junior high school and below	32	15.1
	High school or vocational school	42	19.8
	junior college	48	22.6
	Bachelor’s degree or higher	90	42.5
Occupational status			
	In school	9	4.2
	Employed	144	67.9
	Others	59	27.8

### 3.2 Translation and cultural adaption

#### 3.2.1 Translation results

The C-GUTs was modified in terms of the expression and structure of 15 items to achieve the same meaning and equivalence. After modification, the C-GUTs demonstrated improved clarity and readability. Additionally, the structure of some items was refined to enhance their logical flow and coherence, making the scale more user-friendly for the target population.

#### 3.2.2 Cultural adaption

The process of cultural adaption involved several key steps to ensure that the scale is linguistically accurate and culturally relevant, to make the scale appropriate for the Chinese context. Firstly, a group of experts was assembled to review the translated items. Their expertise helped identify any cultural nuances or potential misunderstandings that might arise from a direct translation. Secondly, a small sample of patients with IBS was tested using C-GUTs as pilot study to gather feedback on its cultural appropriateness and ease of understanding. Based on the results, the wording and expression of some entries were fine-tuned to better align with Chinese cultural norms and values. The result of the Panel experts reviewing: the expert’s active degree was 100.0%, the authority coefficient was 0.879, the semantic, idiom and concept of 15 items were revised to make them consistent with the expert’s opinion. The Result of pilot testing: a total of 45 investigating forms was dispatched and returned, and the valid rate was 100%. The time of filling out the questionnaires was 2.7–7.8 min, the average time was 3.5 ± 1.6 min. All the investigated patients thought that the language of the C-GUTs was appropriate, easy-understanding, no ambiguity and clear.

### 3.3 Psychometric validation

#### 3.3.1 Item analysis

Item analysis method was employed to evaluate the discriminative power of each item. An independent samples *t*-test was used to calculate the critical ratio (CR) by comparing the scores of the top 27% and bottom 27% of participants. Items with *p* ≥ 0.05 were considered for deletion. The results indicated that the significance corresponding to each *t*-value was <0.05, and the absolute values of the *t*-value are greater than 3, suggesting a significant difference between the high-score group and the low-score group for this item. The CR values are valid, demonstrating that all items possess high discriminative power. Analysis of the relationship between items and the total scale score using Pearson correlation revealed that correlation coefficients ranged from 0.413 to 0.790 (all *P*-values were <0.01), all exceeding 0.4. This confirms that all items exhibit high discriminative validity.

#### 3.3.2 Validity assessment

##### 3.3.2.1 Content validity

The I-CVI and S-CVI calculated during the expert committee review were reported. The C-GUTs illustrated I-CVI ranging from 0.8 to 1.0, with S-CVI and the average level of C-GUTs being 0.80 and 0.987, respectively, indicating favorable content validity of C-GUTs.

##### 3.3.2.2 Construct validity

The KMO result was 0.925, and Bartlett sphericity test (approximate χ^2^ = 2437.512, *P* < 0.001) indicated that the sample scores were appropriate for factor analysis. The initial eigenvalues showed that the initial factor accounted for 57.7% of the variance, the second factor accounted for 8.5%, and the third factor accounted for 6.2%. However, the pattern matrix and scree plot of the initial eigenvalues suggested that only two factors were appropriate. Subsequently, repeated two-factor analysis was conducted until all included items met the criteria. In the final version of C-GUTs, the first factor (reflecting PC) and the second factor (reflecting VS) accounted for 57.7% and 8.5% of the total variance, respectively, with a correlation of 0.78 between them.

###### 3.3.2.2.1 Exploratory factor analysis

Kaiser-Meyer-Olkin test and Bartlett’s test of sphericity were conducted to assess the sampling sufficiency. Principal axis factoring combined with varimax rotation was employed for factor extraction. Principal axis factoring was employed for factor extraction (29). This method focuses on analyzing the common variation among variables, aiming to identify potential common factors rather than merely explaining the total variance. Subsequently, orthogonal rotation was conducted using the Varimax method to ensure mutual independence among factors and obtain clear factor structures (30). The number of factors was decided by eigenvalues > 1, scree plot inspection, as well as theoretical consistency.

The scale’s KMO value was 0.925, and Bartlett’s test of sphericity yielded an approximate chi-square value of 2437.512 (*P* < 0.001), indicating the feasibility of factor analysis. Principal component analysis with varimax rotation extracted two common factors with eigenvalues greater than 1, accounting for a cumulative variance contribution rate of 66.240%. After varimax rotation, in the factor matrix, the factor loadings of each item in C-GUTs ranged from 0.578 to 0.807 for PC and from 0.578 to 0.837 for VS, all exceeding 0.4 in Table 2.

**TABLE 2:** Factor loading analysis of 15 Gastrointestinal Unhelpful Thinking Scale (GUTs) items based on principal axis factor extraction and orthogonal rotation.

Items	Factors	
	Pain catastrophizing scale	Visceral sensitivity scale
My gastrointestinal discomfort is the most anxiety-inducing thing in my life	0.807	–
I always pay attention to the feeling of gastrointestinal discomfort	0.783	–
I would feel anxious if I didn’t know where the nearest toilet is	0.751	–
I often suffer from gastrointestinal distress and find it hard to get away from it	0.728	–
My gastrointestinal discomfort has been making me anxious	0.719	–
When I am anxious, I worry that my gastrointestinal discomfort will get worse	0.701	–
I’m worried that my digestive tract pain will never get better	0.695	–
My gastrointestinal pain is causing me serious psychological stress	0.639	–
I can’t control the negative thoughts about my stomach and intestines	0.637	–
All I can think of is how to make my stomach and intestines stop hurting	–	0.837
I’m worried that my stomachache could lead to disastrous consequences	–	0.837
My gastrointestinal pain makes it difficult for me to concentrate.	–	0.812
My gastrointestinal pain is causing me to have negative thoughts.	–	0.763
My gastrointestinal pain reminds me of the problems I am facing	–	0.725
My stomach and intestinal pain makes me very angry	–	0.578

###### 3.3.2.2.2 Confirmatory factor analysis

**FIGURE 2:**
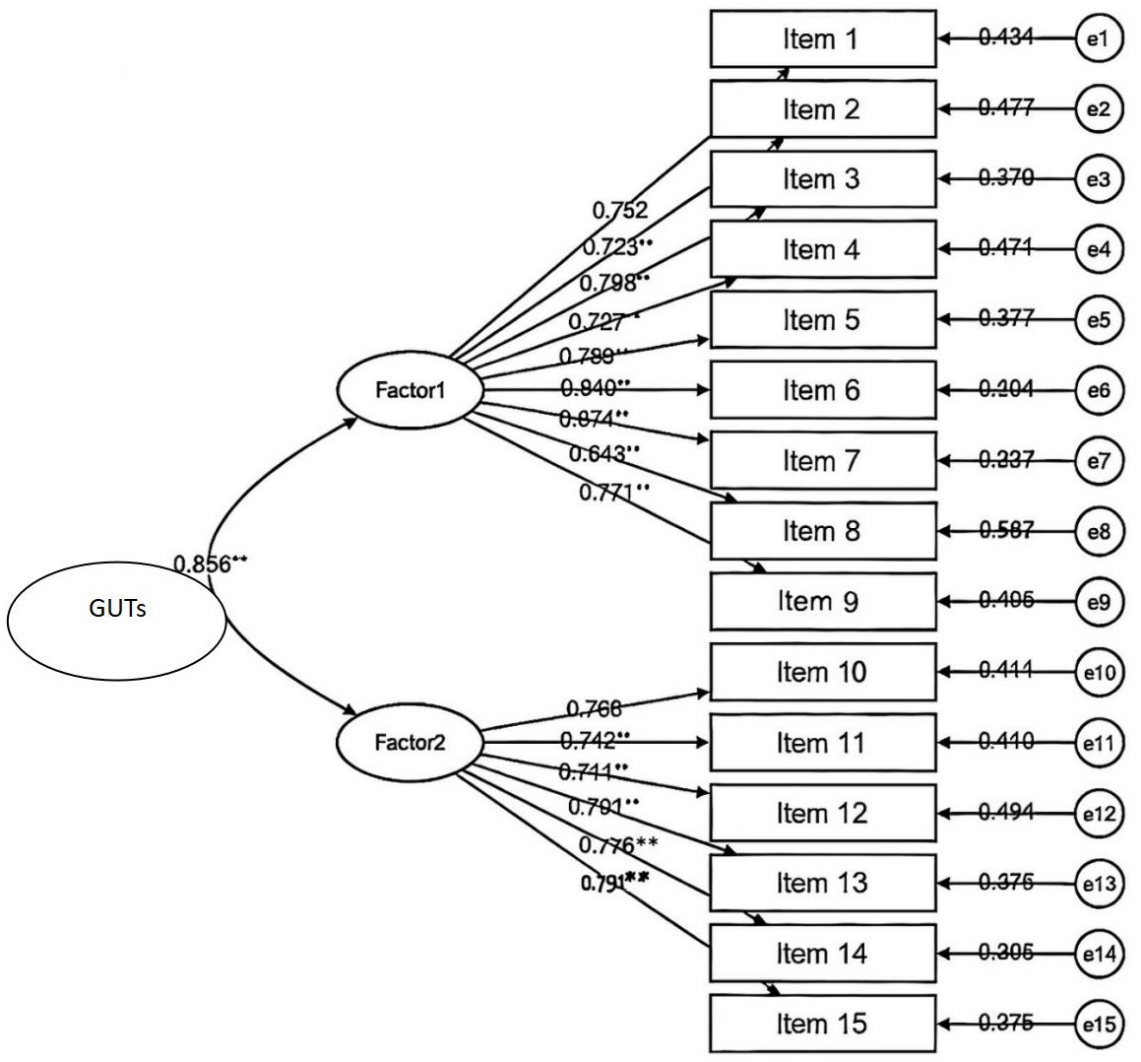
Confrmatory factor analysis of the Chinese version of Gastrointestinal Unhelpful Thinking Scale (GUTs).

In this study, confirmatory factor analysis was conducted with the scale’s two dimensions as latent variables and 15 items as observed variables to evaluate its construct validity. The results demonstrated good fit with the two-factor model. Specifically, the chi-square freedom ratio (χ^2^/df) of 2.991 was below the threshold of 3, indicating a satisfactory fit. CFI value (0.853) and TLI values (0.826) were both exceeded the recommended threshold of 0.80, confirming acceptable fit. Additionally, RMSEA (0.038) was below the 0.08 critical value, further validating the model’s applicability. These findings collectively confirm the scale’s effectiveness in measuring the intended constructs, seen in Figure 2 and Table 3.

**TABLE 3:** Confirmatory Factor Analysis (CFA) results for the 15-item scale (*N* = 212).

Index for goodness of fit	Standards or thresholds	Results	Fit test
Chi-square ratio, X^2^/df	<2 Good; <3 Average; <5 Fair	2.991	Yes
Goodness-of-fit index, GFI	>0.90	0.972	Yes
Root mean square error of approximation, RMSEA	<0.05 very good; <0.08 good; <0.10 fair	0.038	Yes
Normed fit index, NFI	>0.80	0.824	Yes
Incremental fit index, IFI	>0.80	0.854	Yes
Tucker-Lewis index, TLI	>0.80	0.826	Yes
Comparative fit index, CFI	>0.80	0.853	Yes
Adjust goodness-of-fit index, AGFI	>0.80	0.692	No

#### 3.3.3 Reliability assessment

##### 3.3.3.1 Internal consistency

Cronbach’s α was used for calculation for the total scale and its two dimensions. A value of Cronbach’s ≥ 0.70 was considered acceptable, ≥0.80 being good, and greater than 0.90 being excellent (31). The Cronbach’s α results of the total scale and its two dimensions were 0.947, 0.926, 0.894, respectively. The 95% CI for Cronbach’s α of the total scale was 0.931–0.960, and for the PC and VS subscales were 0.905–0.943 and 0.867–0.917, respectively, which indicated an excellent effect size.

##### 3.3.3.2 Test-retest reliability

The Intraclass Correlation Coefficient (ICC) was calculated using a two-way mixed-effects model for absolute agreement for the total and subscale scores between the two time points. A value of ICC > 0.75 represents good reliability, and >0.90 indicates excellent reliability (25). In this study, the 2-week test-retest reliability for the scale was analyzed using a sample of 20 patients and its ICC values for two dimensions were 0.844, 0.850, 0.813, respectively. The 95% CI for ICC of the total scale was 0.765–0.901, and for the PC and VS subscales were 0.772–0.905 and 0.721–0.885, respectively; all ICC values showed a good effect size (ICC > 0.75), indicating stable test-retest reliability.

##### 3.3.3.3 Split-half reliability

The scale items were split into two halves (odd vs. even items), and the Spearman-Brown and the Guttman coefficient were conducted in this study. A value > 0.80 was considered satisfactory (32). In this study, Spearman-Brown’s split-half reliability of the total scale and two dimensions were 0.912, 0.938, 0.874, respectively, and Guttman’s split-half reliability for the total scale and two dimensions were 0.910, 0.932, 0.874, respectively.

## 4 Discussion

This study presents a comprehensive translation, cultural adaptation, and psychometric validation of the C-GUTs for application in a Chinese population with IBS. The findings strongly support the C-GUTs as a valid and reliable tool for assessing the core cognitive-affective constructs of PS and VS. The rigorous methodological approach, which includes the Brislin translation model, expert consultation, cognitive interviewing, and advanced psychometric testing (EFA, CFA), ensured both linguistic accuracy and conceptual equivalence with the original scale (1, 17, 18).

### 4.1 The scientificity of the Chinese version of GUTs

In this study, the C-GUTs was ensured to be scientifically valid through translation and cultural adaptation. The quality of translation is crucial for cross-cultural measurement tools, as it can determine the quality of the research. Therefore, during the translation phase, this study adopted the Brislin translation model (20), with repeated comparisons to ensure equivalence in culture, function, and content, thereby guaranteeing translation accuracy. During the cultural adaptation period, expert consultations were carried out to revise the scale, conveying the original meaning and reducing cultural disparities, thus enhancing its applicability (18). The results of the translation showed that the C-GUTs maintained a high level of semantic equivalence with the original GUTs. The cultural adaptation process further refined the scale, making it more suitable for the Chinese context. This was evidenced by the positive feedback from the experts who participated in the consultations, indicating that the revised scale effectively captured the intended constructs and minimized cultural biases. The C-GUTs demonstrated good content validity and structural validity. The internal consistency (Cronbach α = 0.947) and split - half reliability (Spearman = 0.912, Guttman’s = 0.910) indicated excellent item homogeneity and scale consistency (27, 32). The excellent ES of reliability coefficients (Cronbach’s α = 0.947, ICC = 0.844) and their 95% CIs excluding the acceptable threshold further confirm that the C-GUTs has stable and reliable measurement properties, and the results are not affected by sampling error. It accurately reflects patients’ psychological experiences of pain catastrophizing and trait visceral anxiety. It is suitable for future related studies aiming to evaluate these thought patterns and link them with variations in gastrointestinal and psychological symptoms, as well as to provide assistance in treatment and care. This indicates that the scale can produce stable and reliable results when used repeatedly over time or across different raters. Overall, the C-GUTs is a valuable tool for researchers and clinicians interested in studying the psychological aspects of gastrointestinal disorders and developing targeted interventions. Therefore, it is worthy of widespread clinical application.

### 4.2 The application of the C-GUTs

The C-GUTs is applicable to adult IBS patients aged 18 and above, excluding those with severe organ failure or mental disorders. This scale is suitable for assessing patients’ pain catastrophizing and psychological perceptions related to visceral sensitivity (17). It effectively meets the strong cognitive needs typical of this age group, demonstrating excellent practicality and operability. The factor loadings of all items showed good to excellent ES, and the cumulative variance explained reached a good effect size (66.240%), which means the C-GUTs can effectively capture the core cognitive constructs of PC and VS in Chinese IBS patients, and the assessment results have clear clinical and psychological meaning. The introduction of the C-GUTs fills a significant gap in the psychological assessment tools available to Chinese healthcare professionals treating IBS.

The C-GUTs holds significant potential for application in various clinical and research settings within the Chinese-speaking population. Firstly, in clinical practice, it can serve as a valuable screening and assessment tool for healthcare professionals, particularly gastroenterologists, psychologists, and primary care physicians, to identify and evaluate the psychological distress and quality of life issues experienced by patients with gastrointestinal disorders (33, 34). By quantifying the psychological impact, clinicians can gain a more comprehensive understanding of the patient’s condition beyond the physical symptoms, enabling the development of more personalized and holistic treatment plans. For example, it can help identify patients with high levels of anxiety or depression related to their GI symptoms, prompting timely referrals to mental health professionals or the integration of psychological interventions into their care.

Secondly, in research contexts, the C-GUTs provides a standardized and validated instrument for investigators to explore the relationships between psychological factors and gastrointestinal disorders in Chinese populations. It can be used in epidemiological studies to estimate the prevalence of psychological comorbidities in patients with specific GI conditions, such as IBS, inflammatory bowel disease, or functional dyspepsia. Furthermore, it can be employed in intervention studies to evaluate the effectiveness of psychological therapies (e.g., cognitive-behavioral therapy, mindfulness-based stress reduction) or combined medical-psychological treatments on improving patients’ psychological wellbeing and overall GI-related quality of life. Longitudinal studies utilizing the C-GUTs can also track changes in psychological status over the course of the disorder or treatment, providing insights into the natural history and prognostic factors of GI disorders from a psychosocial perspective.

Additionally, the C-GUTs can contribute to patient education and communication. By administering the scale and discussing the results with patients, healthcare providers can facilitate open conversations about the emotional aspects of living with a GI disorder, helping patients better understand and cope with their condition. This enhanced communication can strengthen the patient-provider relationship and empower patients to actively participate in their own care.

Moreover, the availability of a validated Chinese version allows for cross-cultural comparisons in GI research, enabling researchers to investigate whether the psychological impact of GI disorders varies across different cultural contexts, including between Chinese and Western populations. This can provide valuable insights into the role of cultural factors in shaping the experience of illness and inform the development of culturally sensitive interventions.

## 5 Strength and limitation

The study conducted a comprehensive approach to evaluating the psychometric properties of the instrument in question, encompassing both validity and reliability assessments. The application of rigorous statistics enhances the robustness of the findings, providing a solid foundation for explaining the results. Additionally, the inclusion of a wide range of potential influencing factors, such as dysfunctional attitudes and coping styles, offers deep insights into the complicated relationship between psychological constructs and gastrointestinal symptoms. However, limitations must also be acknowledged. The cross-sectional design of this study limits the ability to infer causality, and reliance on self-reported measurements may introduce bias. The same 212 participants were used for both exploratory and confirmatory factor analyses without sample splitting or independent validation, which may reduce the generalizability of the factor structure. And the study did not conduct a formal assessment of convergent validity and discriminant validity, which may limit the comprehensiveness of the validity evaluation. Furthermore, the generalizability of the findings may also limited by the certain cultural context of research, highlighting the need for further cross-cultural validation in diverse populations.

## 6 Conclusion

In conclusion, while the study provides deep insights into the psychometric evaluation of the instrument within a specific cultural context, several limitations should be clarified. The cross - sectional design of study hinders definitive statements about causality, and the reliance on self-reported data may cause responsive biases. The cultural specificity of the sample also limits the finding’s generalizability, underscoring the importance of conducting similar studies in diverse cultural settings. Future research should focus on replicating these findings across different populations and employing longitudinal study designs to gain deeper insights into the causal relationships between variables. Additionally, incorporating multiple data sources, such as observer ratings or physiological measures, could enhance the validity of the results. In spite of these limitations, this study contributes to the growing body of cross-cultural psychometric validation literature and lays a foundation for subsequent research in this field.

## 7 Relevance to clinical practice

The translated and validated C-GUTs holds significant relevance to clinical practice in the management of Chinese IBS patients. Firstly, it provides clinicians with a reliable and valid tool to systematically assess gastrointestinal-specific unhelpful thinking patterns. Secondly, the C-GUTs serve as a useful outcome measure in clinical trials evaluating the efficacy of psychological interventions for IBS. Thirdly, the scale can facilitate better communication between patients and healthcare providers. Early identification of unhelpful thinking patterns with the C-GUTs may also enable the implementation of preventive strategies or early interventions to prevent the development of more severe psychological distress or the chronicization of IBS symptoms.

## Data Availability

The raw data supporting the conclusions of this article will be made available by the authors, without undue reservation.
